# Caspase Inhibition Blocks Cell Death and Enhances Mitophagy but Fails to Promote T-Cell Lymphoma

**DOI:** 10.1371/journal.pone.0019786

**Published:** 2011-05-17

**Authors:** Sih-han Wang, Sean M. Martin, Peter S. Harris, C. Michael Knudson

**Affiliations:** 1 Department of Pathology, Roy J. and Lucille P. Carver College of Medicine, University of Iowa, Iowa City, Iowa, United States of America; 2 Free Radical and Radiation Biology Program, Roy J. and Lucille P. Carver College of Medicine, University of Iowa, Iowa City, Iowa, United States of America; Roswell Park Cancer Institute, United States of America

## Abstract

Caspase-9 is a component of the apoptosome that mediates cell death following release of cytochrome c from mitochondria. Inhibition of Caspase-9 with a dominant negative construct (Casp9DN) blocks apoptosome function, promotes viability and has been implicated in carcinogenesis. Inhibition of the apoptosome *in vitro* impairs mitochondrial function and promotes mitophagy. To examine whether inhibition of the apoptosome would enhance mitophagy and promote oncogenesis *in vivo*, transgenic mice were generated that express Casp9DN in the T cell lineage. The effects of Casp9DN on thymocyte viability, mitophagy and thymic tumor formation were examined. In primary thymocytes, Casp9DN delayed dexamethasone (Dex)-induced cell death, altered mitochondrial structure, and decreased oxidant production. Transmission electron microscopy (TEM) revealed that inhibition of the apoptosome resulted in structurally abnormal mitochondria that in some cases were engulfed by double-membrane structures resembling autophagosomes. Consistent with mitochondria being engulfed by autophagosomes (mitophagy), confocal microscopy showed colocalization of LC3-GFP and mitochondria. However, Casp9DN did not significantly accelerate T-cell lymphoma alone, or in combination with Lck-Bax38/1, or with Beclin 1+/− mice, two tumor-prone strains in which altered mitochondrial function has been implicated in promoting tumor development. In addition, heterozygous disruption of Beclin 1 had no effect on T-cell lymphoma formation in Lck-Bax38/1 mice. Further studies showed that Beclin 1 levels had no effect on Casp9DN-induced loss of mitochondrial function. These results demonstrate that neither inhibition of apoptosome function nor Beclin 1 haploinsufficiency accelerate T-cell lymphoma development in mice.

## Introduction

Apoptosis is a highly conserved cell death pathway that regulates tissue homeostasis and normal development. Bcl-2 family members are critical regulators of the apoptosis pathway. The Bcl-2 family can be classified into pro-apoptotic members which includes multi-domain members (Bax, Bak) and BH-3 only members (Bim, Bid), and anti-apoptotic members (Bcl-2, Bcl-xL) [1]. The Bcl-2 family primarily regulates apoptosis through their effects on the mitochondrial outer membrane permeabilization (MOMP). Bax and Bak form a multimeric complex that mediates the release of cytochrome *c*, Smac/Diablo, and other factors from the intermembrane space of mitochondria. In cytosol, cytochrome c binds to Apaf-1 and pro-Caspase-9 forming the apoptosome and activating Caspase-9. This initiator Caspase activates downstream effector Caspases such as Caspase 3/7 that lead to apoptotic cell death. In contrast to Bcl-2, IAP family proteins act downstream of mitochondrial permeabilization as direct inhibitors of Caspase function.

Alterations in apoptosis have been associated with cancer as first illustrated by the overexpression of Bcl-2 in B-cell lymphoma in both human and murine models [2], [3]. Similarly, mutations in Bax and Bim have been found in multiple tumors and can accelerate oncogenesis in murine models [4]–[7]. Alternatively, Bcl-2 family members can paradoxically regulate oncogenesis as illustrated by Bax overexpression increasing T-cell lymphoma (Lck-Bax38/1) [8], [9]. Downstream regulators of apoptosis such as Apaf-1, Caspase-9 and IAP family members have also been examined in oncogenesis. Reduced protein levels of Apaf-1 or Caspase-9 or elevated expression of IAP family members have been found in a variety of cancer cell lines [10]–[12] and primary tumor biopsy samples [13]–[15]. Recent clinical studies report that proteins involved in apoptosome function may provide prognostic information. For example, patients with AML in which the tumor cells express high XIAP levels have reduced survival compared to controls [10]. Similarly, low levels of Apaf-1 were associated with decreased survival in patients with CLL [15].

In mouse studies, Apaf-1 and Caspase-9 deficient mouse embryonic fibroblasts had increased *myc*-induced transformation relative to control cells [11]. However, Apaf-1 or Caspase-9 deficient hematopoietic cells fail to show accelerated *myc*-induced lymphomagenesis [16]. Similarly, a dominant-negative form of Caspase-9 (Casp9DN) interfered with apoptosome function but was unable to cooperate with *myc* to accelerate oncogenesis [17]. However, whether apoptosome inhibition would accelerate oncogenesis outside the context of overexpression of *myc* is unknown.

Autophagy is an evolutionarily conserved lysosome degradation pathway that maintains energy homeostasis and mediates cellular adaptation in response to stress. Defects in the autophagy pathway have recently been associated with cancer formation. Numerous studies suggest that Beclin 1, a rate-limiting autophagy protein, is a haploinsufficient tumor suppressor gene. Beclin 1 is monoallelically deleted in 40–75% of cases of human ovarian, breast, and prostate cancers [18]. Heterozygous disruption of Beclin 1 increased the incidence of sporadic malignancies in mice, including lymphoma [19], [20]. Interestingly, tumor formation in Beclin 1+/− does not involve complete loss of the Beclin 1 protein or gene. Therefore Beclin 1 is considered a haploinsufficient tumor suppressor gene [20]. While the exact mechanisms by which defects in autophagy promote cancer are not known, one model is that inefficient clearance of dysfunctional proteins and/or mitochondria increases oxidative stress and promotes DNA damage and genetic instability [21]–[23].

Selective elimination of dysfunctional mitochondria by the autophagy pathway is termed mitophagy. While the signals that control and regulate mitophagy are largely unknown, two recent studies report that inhibition of the apoptosome may result in mitophagy. Molecular or chemical inhibition of Caspase activity combined with an apoptotic signal, results in cells with impaired mitochondrial function and reliance on glycolysis for cell survival [24]. Overexpression of glyceraldehyde-3-phosphate dehydrogenase (GAPDH) elevates ATP levels and enhances mitophagy in those cells [25]. Whether Beclin 1 plays a rate-limiting role in the activation of mitophagy following MOMP is not clear. Furthermore, the *in vivo* importance of mitophagy in tumorigenesis remains unknown and requires further investigation.

In summary, while numerous studies have suggested that inhibition of the apoptosome may promote mitophagy as well as oncogenesis, this hypothesis has not been rigorously tested in an animal model. To directly test this model, we have developed mice that express a dominant negative form of Caspase-9 in the T cell lineage (Lck-Casp9DN). Herein we demonstrate that apoptosome activity is effectively inhibited and mitophagy increases in thymocytes from these mice. To examine the oncogenic potential of this transgenic line, we determined the rate of tumor formation in Lck-Casp9DN mice crossed to Lck-Bax38/1 and Beclin 1+/− mice, two tumor-prone strains in which altered mitochondrial function has been implicated in oncogenesis.

## Results

### Casp9DN delays cell death and impairs apoptotic pathway in primary thymocytes

**Figure 1:**
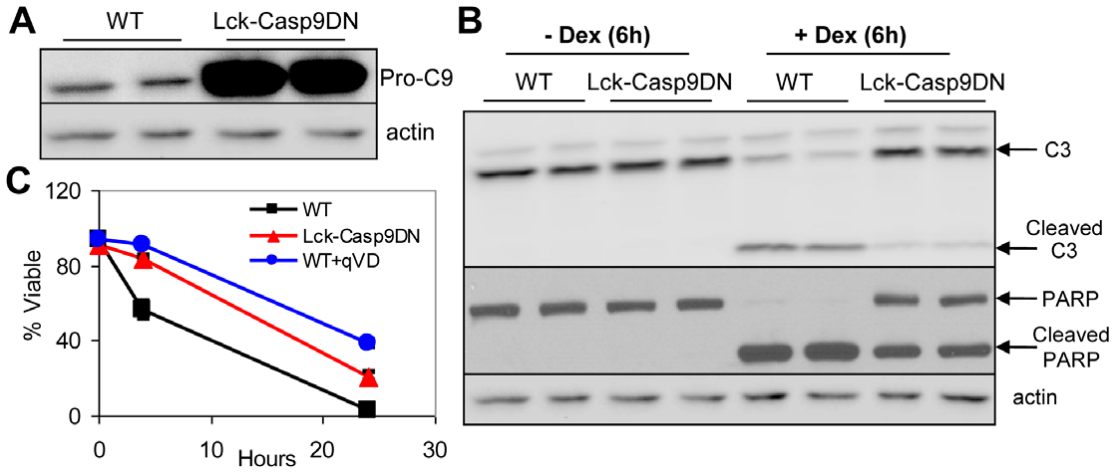
Casp9DN expression inhibits Dex-induced apoptotic cell death in primary thymocytes. **A**) Whole cell lysates were prepared from primary thymocytes from wild type (WT) or Lck-Caspase-9DN (Lck-Casp9DN) transgenic mice as described under “Experimental Procedures”. Immunoblots for pro-Caspase-9 (pro-C9) and actin as a loading control are shown. **B**) Primary thymocytes were isolated from mice of the indicated genotypes, and treated with or without Dex (1 µM) for 6 h. Immunoblots for Caspase-3 (C3), PARP, and actin as a loading control are shown. **C**) Isolated thymocytes from mice of the indicated genotypes were treated with Dex (1μM). Q-VD-OPh (50 μM), a pan Caspases inhibitor, was used as positive control for Caspase inhibition. Cell viability was determined over time by using a Guava flow cytometer and Viacount reagent. The means ± SD for at least duplicate samples are shown.

In order to examine the effect of Caspase-9 inhibition on T cell death and lymphoma formation *in vivo*, transgenic mice were generated using the Lck promoter that directs expression in mature and immature T cells. A high expressing transgenic line was identified and shown to express high levels of Casp9DN with marked inhibition of Caspase-3 and PARP cleavage (Figure 1A and 1B). To determine if Casp9DN delays lymphoid cell death, cell viability was measured in primary thymocytes isolated from wild type or Lck-Casp9DN transgenic mice following Dex treatment (Figure 1C). Q-VD-OPh, a broad-spectrum inhibitor of Caspase activity, was used as a positive control. Expression of Casp9DN markedly reduced cell death following Dex treatment almost as effectively as Q-VD-OPh. These results demonstrate that Casp9DN expression significantly delays *in vitro* apoptotic cell death in thymocytes.

### The effect of Casp9DN on thymic cellularity and proliferation

**Figure 2:**
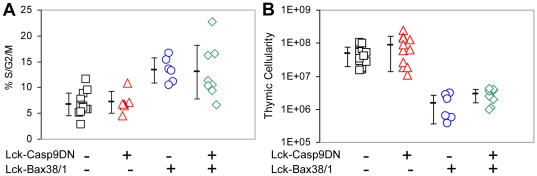
Casp9DN expression has no effect on thymic cellularity and proliferation in Lck-Bax38/1 mice. **A**) DNA content analysis was performed on freshly isolated thymocytes from mice of the indicated genotypes between 8 and 10 weeks of age. Each symbol corresponds to the percentage of cycling cells (%S/G2/M) observed from an individual mouse. The mean ± SD of at least 6 mice per group is shown. **B**) Total thymic cellularity from mice of the indicated genotypes between 8 and 10 weeks of age is shown. Each symbol corresponds to the total viable thymocytes number observed from an individual mouse. The mean ± SD of at least 6 mice per group is shown.

Previous studies suggest that Caspase cascade blockage results in cell cycle arrest following Q-VD-OPh treatment in FL5 cells [24]. Thymic cellularity is markedly reduced and thymocyte proliferation is increased in Lck-Bax38/1 transgenic mice [8]. To determine the effect of Casp9DN on thymus size and thymocyte proliferation, thymocytes were harvested, counted and analyzed for cell proliferation using DNA content analysis for both control and Lck-Bax38/1 mice. Surprisingly, Casp9DN had no effect on thymic proliferation in either control or Lck-Bax38/1 mice (Figure 2A). If Casp9DN effectively blocked apoptosis *in vivo*, we hypothesized that cellularity would be increased in Lck-Casp9DN transgenic mice. However, for both control and Lck-Bax38/1 mice, thymic cellularity was unaffected by Casp9DN expression (Figure 2B). These results suggest Casp9DN expression had little effect on the survival of thymocytes *in vivo* with or without Bax expression. However, these results examining whole organ homeostasis, do not rule out the possibility that a small fraction of cells are protected from cell death for a short period of time following a death signal.

### Isolated thymocytes from Lck-Casp9DN transgenic mice have reduced ROS levels

**Figure 3:**
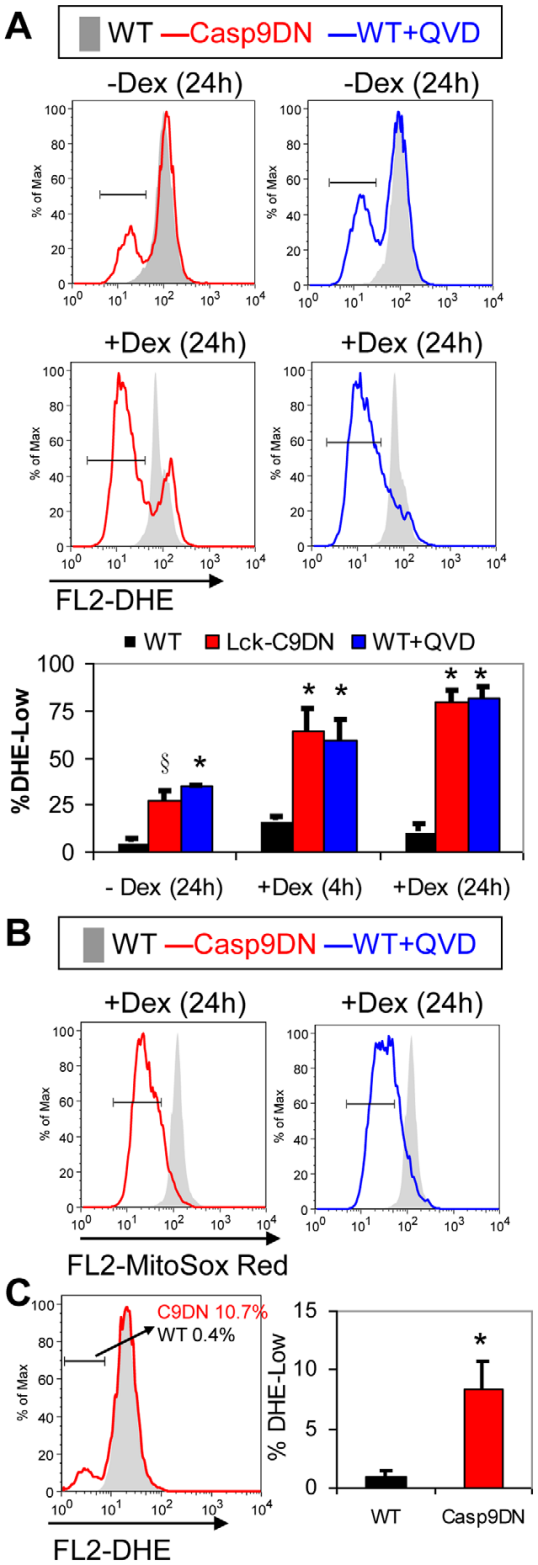
Casp9DN reduces ROS levels in primary thymocytes. **A**) Representative histogram overlays of DHE stained viable thymocytes is shown. All cells were treated with or without Dex as indicated. Caspase activity was inhibited by either Casp9DN expression (red line, Casp9DN) or Q-VD-OPh treatment (blue line, WT+QVD) and compared to control cells (gray shaded, WT-wild type thymocytes). For the histogram overlay graphs, the viability of thymocytes was as follows; 24 h culture without Dex: 46% (WT), 56% (Casp9DN), and 69% (WT+QVD); 24 h culture with 1μM Dex: 7.5% (WT), 20% (Casp9DN), and 42% (WT+QVD). Quantitation of the percentage of cells with low DHE staining (gate shown in histograms) from the samples as indicated is shown in the bottom panel. The mean + SD of at least duplicate mice per group is shown. § p<0.05 versus the control. * p<0.01 versus the control. B) Representative histogram overlays of the MitoSOX Red stained viable thymocytes as indicated is shown. Data is representative of at least 3 independent animals. C) Representative histogram overlays of the DHE staining of the viable freshly isolated thymocytes from wild type mice (gray shaded, WT) and Lck-Casp9DN mice (red line, C9DN) is shown. The numbers are the percentage of cells found within the indicated gate for each sample. Quantitation of % DHE-Low population in freshly isolated thymocytes from mice of the indicated genotypes is shown. The mean + SD of at least 4 mice per group is shown.* p<0.005.

Previous studies show that Casp9DN or Q-VD-OPh treatment reduced reactive oxygen species (ROS) levels following an apoptotic signal [24]. To determine if Casp9DN affects oxidative stress *in vivo*, dihydroethidium (DHE) and MitoSOX Red staining were performed on primary thymocytes with or without Dex treatment. Under these conditions, control cells were treated with Q-VD-OPh so that both chemical and molecular inhibitors of Caspases were examined. Cultured thymocytes die spontaneously in culture and the death is greatly accelerated by Dex treatment. Casp9DN expression or Q-VD-OPh treatment showed a large fraction of cells with reduced DHE staining that was not detected in cells in which caspase activity is not inhibited (Figure 3A). These results were supported by also staining cells with MitoSOX Red, another redox-sensitive reagent that is concentrated in the mitochondria [26]. Casp9DN expressing and Q-VD-OPh treated thymocytes were found to have reduced MitoSOX Red fluorescence indicative of reduced oxidation (Figure 3B). Finally, to determine if these redox changes occur *in vivo*, DHE staining of freshly isolated thymocytes from Lck-Casp9DN transgenic mice also demonstrated a significant population of cells with reduced DHE staining (Figure 3C).

### Caspase inhibition alters mitochondrial morphology in primary thymocytes and FL5 cells

**Figure 4:**
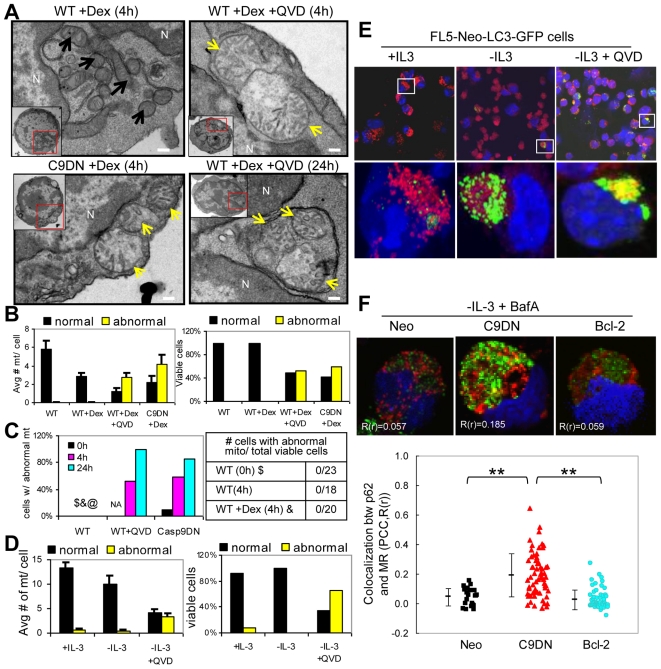
Caspase inhibition increases abnormal mitochondria and activates mitophagy. **A**) TEM images showed morphologic changes in mitochondrial structures (normal mitochondria marked with black arrows and abnormal mitochondria marked with yellow arrows) in isolated thymocytes from mice of the indicated genotypes following the indicated treatment. One of examples of mitophagy showed that a mitochondrial cluster was engulfed within double-membrane autophagosome in bottom-right image. Four images have the same magnification. Scale Bar  = 0.2 µm. N = nucleus. **B**) Quantitation of the average number of normal and abnormal mitochondria per viable cell (left panel) and the percentages of cells with at least 1 normal mitochondrion or with at least 1 abnormal mitochondrion (right panel) in isolated thymocytes from mice of indicated genotypes following 4 h of the indicated treatment. **C**) Quantitation of the percentage of cells with at least 1 abnormal mitochondria in isolated thymocytes from mice of the indicated genotypes following various times of Dex treatment. Table shows that no abnormal mitochondria were observed in WT thymocytes following various treatments. In the WT thymocytes treated with 24 h of Dex, 5% of the cells were viable, so this sample is not shown (@). WT thymocytes treated with 0 h of Q-VD-OPh were not available (NA). **D**) In FL5-Neo cells, quantitation of the average number of normal and abnormal mitochondria per viable cell (left panel) and the percentages of viable cells with at least 1 normal mitochondrion or with at least 1 abnormal mitochondrion (right panel) following 24 h of the indicated treatments. **E**) FL5-Neo cells transfected with LC3-GFP (green) were stained with MitoTracker Red (red, MR) for mitochondria and TO-PRO-3 probe (blue) for nuclear counter staining following 48 h of the indicated treatment. The viability of FL5 cells: 97% (+IL-3), 9% (-IL-3), and 66% (-IL-3+QVD). Colocalization of MR and LC3-GFP indicated mitophagy activation. **F**) FL5 cells with the indicated expression were immuno-stained with p62 (green), MR (red) for mitochondria and TO-PRO-3 probe (blue) for nuclear counter staining following 48 h of IL-3 deprivation and BafA (20 nM). Pearson's correlation coefficient (PCC, R(r)) which statistically measures colocalization of p62 and MR is shown in each image. Quantitation of PCC of the treated FL5 cells is shown. Each symbol corresponds to PCC of each viable cell of the indicated cell lines. The mean ± SD of at least 22 cells per cell line in each treatment is shown. ** p<0.0001.

To determine the effect of Caspase inhibition on mitochondrial morphology, TEM was performed on thymocytes following various treatment conditions. In the absence of Caspase inhibition, all viable thymocytes had mitochondria with well-defined cristae, intact and clear double membranes, homogenous density and cylindrical shape. In contrast, thymocytes expressing Casp9DN or treated with Q-VD-OPh frequently had abnormal mitochondria with disorganized cristae, a discontinuous double membrane, decreased electron density and an increase in the minor axis consistent with mitochondrial swelling (Figure 4A). Importantly, individual cells had uniform mitochondria that were either all classified as normal or abnormal. Using these criteria, quantitative analysis after 4 hours of Dex treatment showed that Casp9DN expression or Q-VD-OPh treatment markedly increased the average number of abnormal mitochondria per cell and the percentage of viable cells with abnormal mitochondria was about 50% (Figure 4B). Of note, this percentage is comparable to the level of protection by Caspase inhibition (Figure 1C) and to the percentage of viable cells with low-DHE staining (Figure 3A). Following longer Dex treatment with Caspase inhibition (24 h), the percentages of viable cells with abnormal mitochondria was further increased (Figure 4C). Importantly, Lck-Casp9DN cells fixed immediately following isolation (0 h) had a significant proportion of cells with abnormal mitochondria (Figure 4C), which also closely matched the percentage of freshly isolated thymocytes with low-DHE staining (Figure 3C). TEM was also applied in FL5 cells, an IL3-dependent pro-B lymphocytic cell line. IL-3 deprivation combined with Caspase inhibition significantly increased the average number of abnormal mitochondria per cell and the percentage of viable cells with abnormal mitochondria (Figure 4D). Again, the percentage of cells with abnormal mitochondria was also comparable to the percentage of cells with low-DHE staining (data not shown).

### Caspase inhibition activates mitophagy in FL5 cells and primary thymocytes

Following 24 hours of Dex treatment with Caspase inhibition, we observed abnormal mitochondria engulfed within a double-membrane vesicle that appeared very similar to an autophagosome and consistent with activation of mitophagy (Figure 4A-right bottom). To verify if Caspase inhibition activates mitophagy in lymphoid cells, IL-3 deprived FL5-Neo-LC3-GFP cells were co-stained with MitoTracker Red (MR) and examined with confocal microscopy. In the absence of Caspase inhibition, MR staining did not significantly overlap with LC3-GFP. However, in the presence of Q-VD-OPh, MR staining colocalized with LC3-GFP, indicating the activation of mitophagy in FL5-Neo-LC3-GFP cells (Figure 4E). p62/SQSTM is an adaptor protein that binds to polyubiquitinated proteins and LC3 on the autophagosome membrane to target damaged organelles or unfolded proteins as well as damaged mitochondria to autophagic degradation [23]. To further confirm if mitophagy is specifically activated under the condition of Caspase inhibition, IL-3 deprived FL5 cells were stained with MR, immuno-stained with p62 antibody with green fluorescence, and examined with confocal microscopy. Those cells were also treated with Bafilomycin A1 to prevent p62 degradation and autophagic flux. FL5 cells over-expressing Bcl-2 were also examined. Overexpression of Bcl-2 is shown to prevent MOMP under stress [24] and inhibit autophagy through Beclin 1 interaction [27], [28]. Again, in the condition of Caspase inhibition, MR staining significantly colocalized with the green fluorescence of p62 whereas in the absence of Caspase inhibition or Bcl-2 overexpression, MR staining did not considerably overlap with the green fluorescence of p62 (Figure 4F). Caspase inhibition had no effect on p62 localization in the absence of a cell death signal (data not shown). Furthermore, Pearson's correlation coefficient (PCC, R(r)) was used as standard to measure colocalization between MR and the green fluorescence of p62, indicating mitophagy. Caspase inhibition significantly increased PCC compared to control or Bcl-2 cells (Figure 4F). In sum, these results in FL5 cells and thymocytes demonstrate that apoptotic activation combined with Caspase inhibition results in abnormal mitochondria morphology and the activation of mitophagy.

### Lck-Casp9DN mice are not susceptible to thymic lymphoma

**Figure 5:**
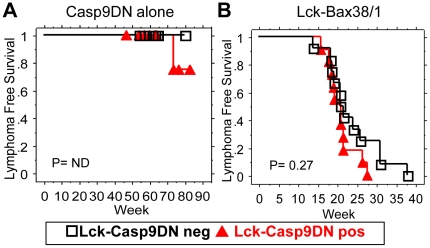
Casp9DN expression does not promote lymphoma formation in Lck-Bax38/1 transgenic mice. Kaplan-Meier analysis for lymphoma free survival **A**) comparing wild type (black open square, N = 8) and Lck-Casp9DN (green solid triangle, N = 12) mice, and **B**) comparing Lck-Bax38/1 (black open square, N = 12) and Lck-Casp9DN x Lck-Bax38/1 (red solid triangle, N = 11) mice. Those mice were followed for lymphoma-free survival as described under materials and methods. P-value of analysis is shown. ND = not determinable without any events in the control group.

To determine the effect of Casp9DN on cancer, lymphoma free survival was examined in Lck-Casp9DN with or without crossing with Lck-Bax38/1 transgenic mice, a strain known to develop thymic lymphomas with a short latency and complete penetrance [8]. Surprisingly, Lck-Casp9DN mice failed to promote oncogenesis either alone or when combined with Lck-Bax38/1 mice (Figure 5). Just a single Lck-Casp9DN mouse had a thymic lymphoma at 74 weeks of age, which was not statistically different from the non-transgenic control group.

### Beclin 1 Haploinsufficiency does not promote T-cell lymphoma formation

**Figure 6:**
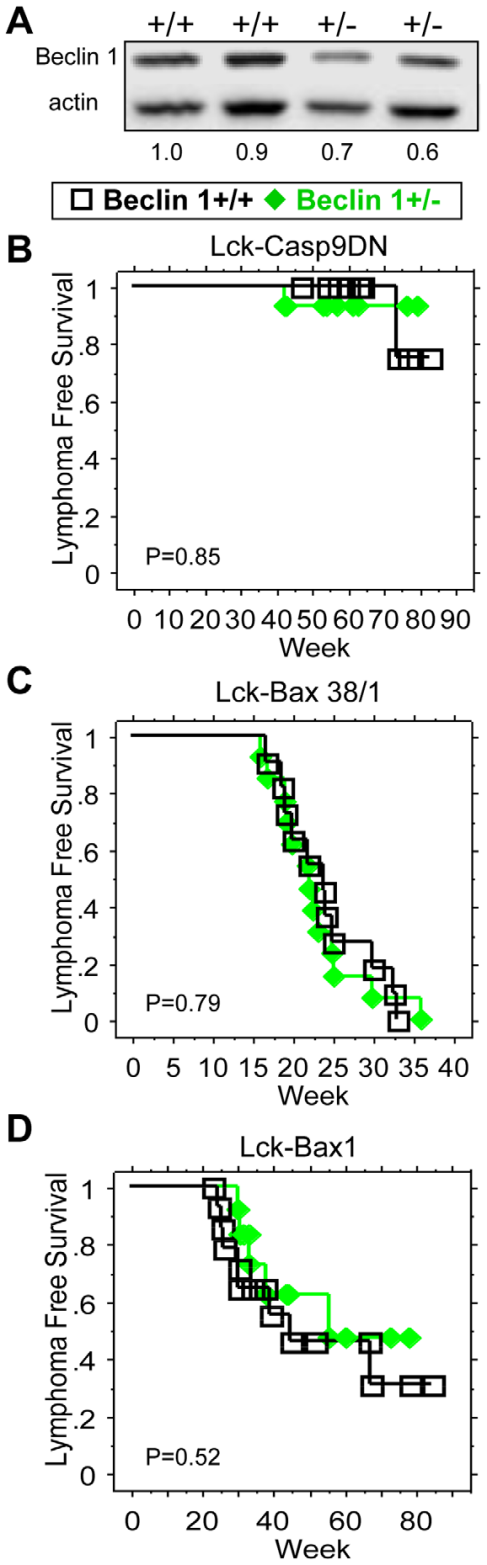
Beclin 1+/− does not accelerate lymphoma formation. **A**) Primary thymocytes were isolated from mice of the indicated genotypes. Immunoblots for Beclin 1, and actin as a loading control are shown. The ratio of Beclin 1 to actin for each mouse is shown below the immunoblots. Kaplan-Meier analysis for lymphoma free survival **B**) comparing Beclin 1+/+ x Lck-Casp9DN (black open square, N = 12) and Beclin 1+/− x Lck-Casp9DN (green solid diamond, N = 14) mice, **C**) comparing Beclin 1+/+ x Lck-Bax38/1 (black open square, N = 11) and Beclin 1+/− x Lck-Bax38/1 (green solid diamond, N = 13) mice, and **D**) comparing Beclin 1+/+ x Lck-Bax1 (black open square, N = 15) and Beclin 1+/− x Lck-Bax1 (green solid diamond, N = 12) mice is shown. Those mice were followed for lymphoma-free survival as described under materials and methods. P-value of analysis is shown.

The results above indicate that Casp9DN expression activates autophagy but failed to accelerate rate of lymphoma development in these models. Autophagy has recently been implicated as an important tumor suppressor pathway based on studies in both mice and humans [18]–[20]. Beclin 1 is a rate-limiting component of the autophagy pathway and has been shown to be a haploinsufficient tumor suppressor [20]. Therefore, we hypothesize that Beclin 1 may play an important role in limiting tumor formation in a Lck-Casp9DN transgenic model. We therefore examined the effect of Beclin 1+/− on tumor development in Lck-Casp9DN, Lck-Bax1, and Lck-Bax38/1 transgenic mice. As expected, thymocytes from Beclin 1+/− mice had reduced Beclin 1 protein levels (Figure 6A). Tumor watch analysis of Lck-Casp9DN mice for up to 90 weeks showed very low penetrance of lymphoma with no significant difference between Beclin 1 wild type and heterozygous mice (Figure 6B). To determine if Beclin 1 would be rate limiting in a model with high tumor penetrance, a tumor watch was performed on Lck-Bax38/1 mice that were either Beclin 1 wild type or heterozygous. Again, no significant difference in tumor formation was observed between Beclin 1 wild type and heterozygous mice (Figure 6C). Finally, to determine if Beclin 1 would be rate limiting in a model with intermediate latency and penetrance, a tumor watch was performed on Lck-Bax1 mice that were either Beclin 1 wild type or heterozygous. Again, Beclin 1 gene dosage had no significant effect on tumor formation in this model (Figure 6D).

### Beclin 1 gene dosage does not affect cell death or ROS levels

**Figure 7:**
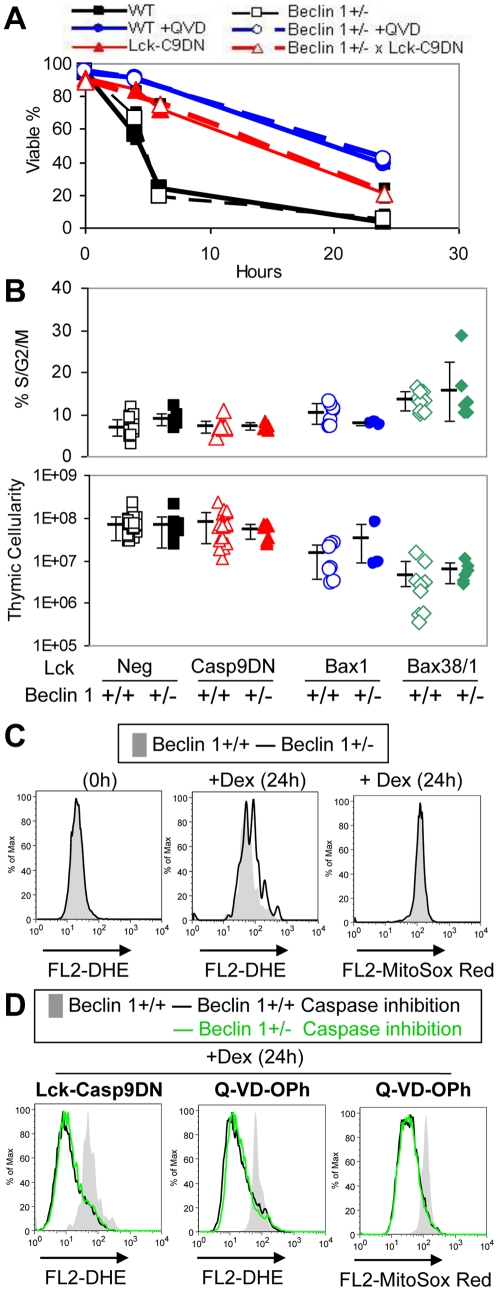
Beclin 1+/− has no effect on cell death, proliferation and ROS levels in thymocytes. **A**) Isolated thymocytes from mice of the indicated genotypes were treated with Dex (1μM), using Q-VD-OPh (50 μM) as positive control for Caspase inhibition. Viability was determined over time. The means ± SD of at least 2 mice per group are shown. B) DNA content analysis of thymocytes (top panel) and thymic cellularity (lower panel) were performed on freshly isolated thymocytes from mice of the indicated genotypes between 8 and 12 weeks of age. Each symbol corresponds to the percentage of cycling cells (%S/G2/M) and the total viable thymocytes observed from an individual mouse. The mean ± SD of each group is shown. C) Representative histograms overlays of DHE and MitoSOX Red staining of the viable thymocytes isolated from Beclin 1+/+ (gray shaded) and Beclin 1+/− (black line) mice following the indicated treatment are shown. The viability of thymocytes following 24h of Dex treatment: 7.3% (Beclin 1+/+) and 8.9% (Beclin 1+/−). These results are representative of at least 3 independent experiments. D) Representative histogram overlays of the DHE and MitoSOX Red staining of the viable thymocytes isolated from Beclin 1+/+ and Beclin 1+/− mice are shown. For each graph, Beclin 1+/+ cells without caspase inhibition (gray shaded), Beclin 1+/+ cells with caspase inhibition (black line) and Beclin 1+/− cells with caspase inhibition (green line) either by Casp9DN expression or Q-VD-OPh treatment are shown. The viability of thymocytes following 24h of Dex treatment was as follows: 7.3% (Beclin 1+/+), 20% (Beclin 1+/+ x Casp9DN), and 22% (Beclin 1+/− x Casp9DN), 42% (Beclin 1+/+ Q-VD-OPh), and 43% (Beclin 1+/− Q-VD-OPh).

Autophagy has been shown to either promote or inhibit cell death. Relevant to cell death regulation, autophagy has been shown to be required for survival in Bax/Bak double deficient cells [29]. We therefore determined if Beclin 1 gene dosage affects cell death in thymocytes with and without Caspase inhibition (Lck-Casp9DN or Q-VD-OPh) following treatment with Dex. Although Caspase inhibition again provided significant protection from cell death, Beclin 1 gene dosage had no effect on cell death under these conditions (Figure 7A). Consistent with this *in vitro* result, Beclin 1 gene dosage had no effect on thymic cellularity and proliferation in control, Lck-Casp9DN, Lck-Bax1, and Lck-Bax38/1 mice (Figure 7B). To determine if Beclin 1 gene dosage affects ROS levels, DHE and MitoSOX Red staining were performed on either freshly isolated thymocytes or cells cultured in Dex. Under all conditions, Beclin 1 gene dosage had no effect on DHE and MitoSOX Red staining (Figure 7C). To determine if Beclin 1 gene dosage affected ROS in cells induced to undergo mitophagy, thymocytes were treated with Dex in the presence of Q-VD-OPh or Casp9DN expression and stained with DHE or MitoSOX Red. Again, Beclin 1 gene dosage had no effect on ROS levels under these conditions (Figure 7D). These results demonstrate that Beclin 1 is not rate limiting in Casp9DN-induced tumor formation in these murine models and does not affect ROS alterations under these various conditions.

## Discussion

Caspase inhibition has been shown to block apoptosome function and reduce apoptotic cell death [30], [31]. Although Caspase inhibition prevents apoptotic cell death, it does not provide significant long-term clonogenic survival (Figure 1C) [24], [25], and most cells eventually die by Caspase-independent cell death pathway. Previous studies show that short-term cell survival with apoptosome inhibition is dependent upon energy supplied by glycolysis [24], [25]. Importantly, recent studies suggest that rare cells that have undergone MOMP may retain proliferative capability. For example, enforced GAPDH expression maintains ATP levels, enhances autophagy and promotes clonogenic survival [25]. More recently, it is shown that MOMP within a single cell is sometime incomplete, leaving some mitochondria intact and permitting clonogenic survival [32]. However, all those studies were based on *in vitro* examinations. Whether cells can recover from MOMP and whether this event would promote oncogenesis *in vivo* is unclear.

There is conflicting data related to inhibition of Caspase-9 and/or apoptosome function and cancer. For example, numerous studies of human cancer have demonstrated that Apaf-1 or Caspase-9 are commonly reduced in cancer [11], [12], [15]. However, mutations in either Caspase-9 or Apaf-1 have not been commonly identified in cancers [33], [34]. Similarly, conflicting results have been reported utilizing murine models. Apaf-1 or Caspase-9 deficient mouse embryonic fibroblasts cooperated with *myc* to promote oncogenic transformation in nude mice [31]. However, in a *myc*-induced lymphoma model, Caspase-9 or Apaf-1 deficiency had no effect on the rate of tumor onset [16]. In sum, whether regulators of the apoptosome are involved in oncogenesis remain unknown.

Here, we generated an *in vivo* model of Casp9DN expressed in the T cell lineage and examined its effect on oncogenesis. Similarity to our previous *in vitro* study [24], Casp9DN significantly inhibited apoptosome function in thymocytes (Figure 1B). Following *in vitro* Dex treatment, we observed a population of Casp9DN-expressing thymocytes with abnormal mitochondria and reduced DHE and MitoSOX Red oxidation. TEM images revealed that Casp9DN cells with dramatically altered mitochondrial morphology and apparent localization within autophagosomes consistent with increased mitophagy. Importantly, about 5–10% of freshly isolated thymocytes from Lck-Casp9DN mice displayed reduced DHE oxidation indicative of altered mitochondrial function. TEM studies of freshly isolated thymocytes from Lck-Casp9DN mice confirmed that some of these cells had altered mitochondrial morphology. These results demonstrate that thymocytes *in vivo* are protected from MOMP-dependent death by Casp9DN. However, Casp9DN expression had no effect on thymic cellularity and proliferation *in vivo* suggesting that cellular homeostasis is not significantly altered in these mice. However, since oncogenesis is thought to occur following multiple mutations within a single cell, we proceeded to determine whether Casp9DN expression alone or in combination with other proven oncogenic models would promote oncogenesis. Over the first year of the tumor watch, none of the control or Casp9DN animals developed evidence of lymphoma. When the study was terminated and post mortem necropsies performed, one Casp9DN animal that was nearly 1.5 years-old had developed a thymic lymphoma. It is possible that additional thymic tumors would have been observed had the study continued for the entire life of the mice. However, it is clear that Casp9DN expression in the thymus of mice did not increase thymic lymphoma formation in the first year of life.

In addition, we also determined whether Casp9DN would promote oncogenesis in two other cancer-prone strains. Lck-Bax38/1 mice were examined since lymphoma formation is thought has been shown to be regulated in part by mitochondrial derived pro-oxidants [35]. In addition, since mitophagy was activated in Lck-Casp9DN mice, we also examined oncogenesis in tumor-prone mice with impaired autophagy (Beclin 1+/−). Surprisingly, Casp9DN had no effect on the rate of tumor development in either of these models. These data extend the previous studies that have demonstrated that Caspase-9 or Apaf-1 deficiency failed to promote oncogenesis in a *myc*-induced lymphoma model [16], [17]. We conclude that inhibition of the apoptosome does not significantly promote lymphoma formation in mice. These studies have not addressed whether Casp9DN would promote epithelial tumor formation. Given that many known oncogenes and tumor suppressor genes regulate T-cell lymphoma in mice, we predict that inhibition of the apoptosome will not significantly alter tumor formation in epithelial cells. In addition, the role of apoptosome inhibition in the formation of neuronal tumors is not addressed. Importantly, previous studies demonstrate that inhibition of Caspases and/or the apoptosome provides long-term protection from death of neuronal cells [30]. This is true *in vivo* in a stroke model [36] as well as *in vitro* models utilizing primary cultures [37], [38]. While other studies would be required to definitively address the role of the apoptosome in neuronal tumor formation, one study failed to find loss of function mutations in Caspase-9 in neuroblastoma [39].

Beclin 1 is an essential autophagy gene for early embryonic development and reported to be a haploinsufficient tumor suppresser gene based on genetic studies in human cancer samples as well as gene heterozygous deletion studies in mice [18]–[20]. Recently, *in vitro* studies suggest that loss of one allele of Beclin 1 leads to elevated ROS levels, increases DNA damage and chromosomal instability, and promotes tumor growth in nude mice [21]–[23]. However, the cell lines used in these studies were genetically manipulated to inactivate cell cycle checkpoints and to block apoptosis. Thus whether Beclin 1 haploinsufficiency would cooperate with other pro-oncogenic pathways and promote oncogenesis in an intact organism has not yet been investigated. Since ROS and chromosomal instability have been implicated in oncogenesis in Lck-Bax38/1 mice, we examined whether Beclin 1 gene dosage would affect tumorigenesis in this model. Surprisingly, Beclin 1+/− did not significantly promote T-cell lymphoma formation in Lck-Bax38/1 mice. It is possible the Beclin 1+/− had no affect in Lck-Bax38/1 mice due to the high penetrance and rapid tumor development in these mice. Therefore, we also examined the effect of Beclin 1 gene dosage in Lck-Bax1 mice, which develop lymphoma with only about 50% penetrance at one year. Again, our results show that Beclin 1+/− did not affect lymphoma formation in Lck-Bax1 mice. Finally, we examined whether Beclin 1+/− would cooperate with Casp9DN to promote oncogenesis. Again, over the 90 weeks of the study, no difference in lymphoma free survival was observed in the Beclin 1+/− mice. These results are consistent in the previous Beclin 1+/− studies in which tumor development was determined only after necropsy. In our study, we only examined the mice for overt thymic lymphomas as this was the site of expression of Casp9DN. Tumor formation in other organs was not systematically determined. Again Beclin 1 gene dosage had no effect on tumor formation under these conditions. In addition to tumor formation, we also directly examined the effect of Beclin 1 gene dosage on ROS levels. Beclin 1 gene dosage did not affect the ROS levels either in directly isolated primary thymocytes or after 24 hours of *in vitro* Dex treatment. Interestingly, Beclin 1 gene dosage had no affect on the increased ROS levels observed in Lck-Bax38/1 thymocytes (data not shown). Finally, Beclin 1 levels did not alter the reduced ROS levels observed in primary thymocytes following either molecular or chemical inhibition of the apoptosome. In sum, our data suggest that Beclin 1 had no effect on ROS levels in primary thymocytes under either basal or stressed conditions. These results suggest that Beclin 1 is not a rate limiting tumor suppressor gene in murine thymic lymphoma.

## Materials and Methods

### Mice

The Lck-Casp9DN construct was generated by ligation of the Casp9DN fragment from polymerase chain reaction (PCR) with a forward primer (5′-GACTAGATCTCCATGGACGAGGCGGACCGG-3′) and reverse primer (5′- GACTAGATCTTCATGAAGTTTTAAAAAACAGCT -3′) of pIRES-GFP-Casp9DN (a gift from Scott Lowe [17]) into the BamHI site of the Lck-hGH vector. This construct contains an inactivating mutation in the active site cysteine that results in inhibition of the endogenous enzyme due to the multimeric nature of the apoptosome. The mice were generated by the University of Iowa Transgenic Facility. In brief, pronuclei from fertilized oocytes (B6/SJL F1 hybrid) were injected with linearized DNA and the oocytes incubated overnight before transplantation into the oviduct of pseudopregnant mice. Ten independent lines were generated and the two lines showing the highest level of expression were chosen for subsequent analysis. The line with the highest level of expression (Line H) was used in this study. Lck-Casp9DN transgenic mice were genotyped by PCR using a forward Caspase-9 cDNA primer (5′-GGCATACACCCTGGATTCGG -3′ within exon 4 of Caspase-9) and a reverse Caspase-9 cDNA primer (5′-AGAGGATGACCACCACAAAGC-3′, within exon 5 of Caspase-9). The primers produced a 270 bp fragment specific to transgene mice or a 520bp fragment in non-transgenic mice after 35 cycles (94°C ×1 min; 55°C ×1 min; and 72°C ×1.2 min). Lck-Casp9DN mice were backcrossed onto a C57BL/6 background and crossed to Lck-Bax38/1 transgenic or Beclin 1 +/- mice for tumor development studies.

Transgenic Lck-Bax mice were previously described and genotyped by PCR method [8]. Lck-Bax38/1 are mice that harbor two independent transgenic Bax constructs as previously described [9]. Lck-Bax1 are mice that harbor one of two independent transgenic Bax constructs as previously described [9]. Beclin 1+/- mice were provided by Dr. Zhenyu Yue (Mount Sinai School of Medicine, The Rockefeller University, NY), and genotyped by PCR, using a forward primer (Neo: 5′- CGCCTTCTATCGCCTTCTTGACGAGTTCT-3′) for Neo, a forward primer (BCNKOT-2R; 5′- GAGCTGGCTCCTGTGAGTATG-3′), and a reverse primer (BCNKOT-1C: 5′- TGGAGGGCAGTCCATACCCTGG-3′) following Dr. Yue's protocol [20]. Beclin 1+/- mice were backcrossed onto a C57BL/6 background and crossed to Lck-Bax1, and Lck-Bax38/1 transgenic mice for tumor development studies.

### Tumor development studies

All mice were maintained in the University of Iowa animal facility under approved protocols by the Institutional Animal Care and Use Committee (IACUC). Mice of the appropriate genotypes were mated to obtain sufficient cohorts of animals needed for the tumor development studies. Littermate controls or controls from the same mating pairs were used for all these studies. Upon entry into the tumor development study, animals were examined weekly for signs of illness or malignancy. Sick animals were monitored more frequently and euthanized when necessary to prevent unneeded suffering. All animals were monitored for up to 90 weeks and then sacrificed for necropsy. Animals that did not show any evidence of lymphoma were censored from the analysis of tumor free survival. When possible, necropsies were performed on dead animals to determine if the animals had gross evidence of lymphoma. Tumors were then confirmed by fixation with 10% formalin and histological examination after H&E staining.

### Cell culturing and viability

Single cells suspensions from thymi were prepared and cell viability by Guava and Annexin V staining were performed as described [24], [40]. Murine FL5.12 hematopoietic prolymphocytic B cells were maintained and deprived of IL-3 for apoptosis induction as previously described [24]. A pan Caspase inhibitor, quinolyl-valyl-O-methylaspartyl-[2,6-difluorophenoxy]- methyl ketone (Q-VD-OPh or QVD, from MP Biomedicals (#03OPH109-03) or SM Biochemicals (#SMPH001)) was used as positive control of Caspase inhibition (50 μM). Dexamethasone (#D1756, Sigma) was used to induce cell death in thymocytes (1 μM). Bafibafilomycin A1 (#B1080, LC Laboratories, 20nM) was used to prevent maturation of autophagic vacuoles by inhibiting fusion between autophagosomes and lysosomes.

### Transfection of FL5 cells with LC3-GFP

FL5-Neo-LC3-GFP cells were generated using a HIV based lentiviral system with the pTZV3 plasmid containing the genes for LC3-GFP and hygromycin resistance as previously described [41]. Initially HEK 293T cells were transfected with Effectene transfection kit (#301425, Qiagen) with the target plasmid pTZV3 Hygro-LC3-GFP. At 18 h post-transfection virus particles were harvested from the HEK 293T cells by collecting the media and filtering with a 0.22 μm syringe filter (Millipore). This collected media was then used to infect the FL5-Neo cells by direct addition to these cells. This transduction procedure was then repeated 4-5 times over a 3-day period. Cells expressing LC3-GFP were selected with 1 mg/mL hygromycin (#10687-010, Invitrogen) for 10 days in culture.

### Immunoblot analysis

Lysates prepared from thymocytes were made in triton buffer (1% triton X-100 in Versene with 20 mM Tris-HCl, pH = 7.5) supplemented with a protease inhibitor cocktail (#P8340, Sigma), phosphatase inhibitor I and II (#P2850 and #P5726, Sigma), and 100 μM PMSF (#P7626, Sigma). Protein concentration was determined using the DC Bradford assay kit (Bio-Rad). Protein (20 μg) was electrophoresed in a 4% to 20% Tris-Glycine Gels (EC60285BOX, Invitrogen), electrotransferred onto a nitrocellulose membrane (Bio-Rad), and then stained with Ponceau S (Sigma) to determine transfer efficiency. The membranes were then blocked in 5% dry fat-free milk in PBST (1(PBS and 0.05% Tween 20) for 1 h at room temperature and probed with primary antibody overnight at 4°C. Primary antibodies against Caspase-9 (#9504), Caspase-3 (#9665), Beclin 1 (#3738) were from Cell Signaling, and actin (#A4700) and PARP (a gift from Joseph J. Cullen) p62/SQSTM1 (#P0067) (a gift from Martha M. Monick) was from Sigma, and GAPDH (#ab9484) was from abcam. After incubation with the primary antibody, the membranes were washed in PBST three times and incubated with the following horseradish peroxidase-conjugated secondary antibodies (1∶10,000) in 5% dry fat-free milk in PBST for 2 h at room temperature: goat anti-rabbit (#L42007, Invitrogen), or goat anti-mouse (#sc-2005, Santa Cruz Biotehcnology). After incubation, the membranes were washed three times in PBST and the proteins identified by ECL (#NEL105001EA, Perkin-Elmer) Densitometry analysis was performed with the UVP Bioimaging System (UVP, Inc., Upland, CA, USA).

### Flow Cytometry Analysis

Flow cytometry analysis was performed on a FACSCAlibur instrument from Becton Dickenson within the University of Iowa Department of Pathology flow cytometry facility. For DNA content analysis, cells were stained with Krishan propidium iodide buffer as previously described [24]. For DHE staining, cells were pelleted and resuspended in PBS containing 5 mM pyruvate, 10 μM DHE (dihydroethidium, #D11347, Invitrogen) and 10 μM antimycin A (#A8674, Sigma) treatment as a positive control. Cells were then incubated for 20 min at 37°C. DHE-stained cells were pelleted and resuspended in annexin V binding buffer with annexin V-Cy5 (1∶1000) (#K103-100, Biovision). Cells were covered and kept on ice until analyzed on a FACSCalibur flow cytometer. Only viable cells (annexin V-negative) were analyzed for DHE fluorescence (FL2). For MitoSOX Red staining, cells were pelleted and resuspended in RPMI media with 5 μM MitoSOX Red (#M36008, Invitrogen) in PBS, and 10 μM antimycin A treatment as a positive control. Cells were then incubated for 10 min at 37°C. MitoSOX Red-stained cells were pelleted and stained with annexin V-Cy5 as described above. Cells were covered and kept on ice until analyzed on a FACSCalibur flow cytometer. Only viable cells (annexin V-negative) were analyzed for MitoSOX Red fluorescence (FL2).

### Microscopic Analysis

All microscopic analysis was performed within the Central Microscopy Research Facility at the University of Iowa. For mitochondrial morphology (TEM), cells were fixed with 2.5% glutaraldehyde in 0.1 M sodium cacodylate (pH = 7.2) and stored at 4°C until embedding. Cells were post-fixed with 1% osmium tetroxide with 1.5% potassium ferrocyanide in 0.1 M sodium cacodylate (pH = 7.2), and then were followed by an increasing gradient dehydration step using ethanol. Cells were then embedded in Spurr low viscosity embedding medium (Ladd Research) and ultra-thin (90 nM) sections were placed on uncoated copper grids, and stained with 0.2% lead citrate and 1% uranyl acetate. Images were examined with a JEM-1230 electron microscope (JEOL, Central Microscopy Research Facilities, The University of Iowa) at 100 kV. For quantitation of mitochondria, the data obtained from a minimum of 25 independent viable cells was averaged (mean ±SD).

For colocalization of mitochondria and LC3-GFP, FL5-Neo-LC3-GFP cells were spun down and resuspended with Mitotracker Red (#M7512, Molecular Probe) in RPMI media (300 nM) for 20 min. Next, cells were washed with PBS twice and resuspended in PBS. Cells were spun onto microscope slides using a cytospin (Cytospin 2, Shandon) with 600× g for 5 min, and were fixed by 4% paraformaldehyde in PBS for 30 min. The slides were then washed 4-5 time with PBS. Cells were nuclear counter stained by TO-PRO-3 (1∶2000 in PBS) for 5 min in the dark. TO-PRO-3 was then removed by wicking away with a paper towel and the coverslips were then mounted onto a slide with 30 µL VECTASHIELD Mounting Medium (#H-1000, Vector Laboratories). Then extra liquid was removed from the coverslip by tilting the slide and wicking away with a paper towel. For colocalization of mitochondria and p62, the cells were stained with Mitotrakcer Red, fixed by 4% paraformaldehyde as described above, and permeabilized for 10 min in 0.2% Triton X-100 in PBS. The slides were then blocked in blocking buffer (PBS, 5% normal goat serum) for 1 h at room temperature. The slides were then incubated overnight at 4°C with polyclonal rabbit anti-p62/SQSTM1 antibody (1∶2000, #P0067, Sigma). The slides were then washed extensively in PBS. The slides were then incubated with Alexa-488-conjugated goat anti-rabbit immunoglobulin (1∶1000) in PBS for 1h at room temperature. Cells were nuclear counter stained by TO-PRO-3 and the coverslips were then mounted onto a slide with VECTASHIELD Mounting Medium as describe above. The samples were imaged with a Bio-Rad MRC 1024 confocal microscope, equipped with a Kr/Ar laser. The LC3-GFP and MitoTracker Red (MR) fluorescence was excited using the 488 nm laser, whereas TO-PRO-3 was excited with the 647 nm laser. For image acquisition, a Bio-Rad MRC 1024 confocal microscope (Central Microscopy Research Facilities, The University of Iowa) equipped with filters for Alexa-488, Alexa-568, and TO-PRO-3 was employed. Final images were prepared using Image J Software. Images were acquired and analyzed with identical settings. Colocalization analysis of Mitotracker Red and green fluorescence of p62 was processed using LaserSharp 2000 software (Central Microscopy Research Facilities, The University of Iowa). The whole area of each live cell with complete nuclear counter staining was margined as region of interest (ROI) and applied to colocalization analysis with the threshold setting in channel 1 (red) and channel 2 (green). Pearson's correlation coefficient (PCC) was used to measure the colocalization of Mitotracker Red and green fluorescence of p62.

### Statistical analysis

Statistical analysis was performed with the StatView Program (SAS Institute Inc.) using Kaplan-Meier cumulative survival to determine if the differences in survival were significant between each of the indicated groups. A two-tailed Student *t* test assuming unequal variance was used to compare differences in cellularity, cell viability, proliferation rate, DHE-Low population, PCC analysis and normalized protein expression between groups.
